# IgA Nephropathy: Current Understanding and Perspectives on Pathogenesis and Targeted Treatment

**DOI:** 10.3390/diagnostics13020303

**Published:** 2023-01-13

**Authors:** Yating Du, Tingzhu Cheng, Chenxuan Liu, Tingting Zhu, Chuan Guo, Shen Li, Xiangrong Rao, Jinpu Li

**Affiliations:** Department of Nephrology, Guang’anmen Hospital, China Academy of Chinese Medical Sciences, Beijing 100053, China

**Keywords:** immunoglobulin A nephropathy, Gd-IgA1, mucosa-associated lymphoid tissue, mucosal immunity, microbiota, complement dysregulation, prognosis, clinical trials, translational researches

## Abstract

Immunoglobulin A nephropathy (IgAN) is the most common primary glomerulonephritis worldwide, with varied clinical and histopathological features between individuals, particularly across races. As an autoimmune disease, IgAN arises from consequences of increased circulating levels of galactose-deficient IgA1 and mesangial deposition of IgA-containing immune complexes, which are recognized as key events in the widely accepted “multi-hit” pathogenesis of IgAN. The emerging evidence further provides insights into the role of genes, environment, mucosal immunity and complement system. These developments are paralleled by the increasing availability of diagnostic tools, potential biomarkers and therapeutic agents. In this review, we summarize current evidence and outline novel findings in the prognosis, clinical trials and translational research from the updated perspectives of IgAN pathogenesis.

## 1. Introduction

Immunoglobulin A nephropathy (IgAN) is the most common primary glomerulonephritis (GN) worldwide, with a high prevalence in Asia (40–50%) and western countries (10–30%) [1]. Although the disease usually runs a benign course, it has been shown that 20–30% of IgAN patients will develop the end-stage renal disease (ESRD) over a period of ten years [2]. There are no disease-specific treatments or early diagnosis strategies for IgAN. Optimal supportive therapy remains the main pillar in IgAN treatment, whereas the efficacy of systemic immunosuppression remains controversial. Clinical marker-based assessments of disease activity, such as serum creatinine, proteinuria and hypertension, is often limited.

At present, a definitive diagnosis of IgAN requires an invasive kidney biopsy demonstrating dominant or codominant mesangial IgA staining. However, a kidney biopsy may result in a delayed diagnosis and potentially poor outcomes. Considering the significant heterogeneity in clinicopathological presentation, disease course and even races in IgAN, early identification of patients at risk of progression and the development of an appropriate treatment plan are essential.

Existing evidence suggests that IgAN is an autoimmune disease with a multi-hit process. It is characterized by abnormal IgA1 synthesis and glycosylation, leading to increased circulating polymeric hypogalactosylated IgA1 (Gd-IgA1). Gd-IgA1-containing immune complexes (Gd-IgA1-ICs) are formed mainly by Gd-IgA1 with specific autoantibodies. The glomerular mesangial deposition of Gd-IgA1-ICs leads to cellular proliferation, activation of complement and subsequent glomerular injury [3]. However, the multi-hit theory cannot fully explain the pathogenesis of IgAN, and the exact production site of Gd-IgA1 remains to be determined [4,5]. It was found that Gd-IgA1 levels were elevated in some asymptomatic first-degree relatives of IgAN patients [6]. On the other hand, asymptomatic Gd-IgA1 deposition is occasionally observed in kidney transplant donors [7,8]. These findings suggest that additional cofactors are required to develop clinically apparent IgAN. Patients with IgAN usually present with hematuria concomitant with mucosal infections in the respiratory and gastrointestinal tracts. Therefore, attention is directed at the exploration of the role of mucosal immune response in IgAN from various aspects, such as genetic susceptibility, foreign antigens and microbiome dysbiosis [9,10,11]. Furthermore, a growing body of evidence suggests that complement activation is associated with glomerular and circulating markers in IgAN. Of great interest, it is postulated that the complement can be the pathogenic contributor or aggravating factor in IgAN [12].

In light of these advances, this review will focus on recent developments in the biomarkers, prognostic tools and potential therapies targeting every key stage in IgAN pathogenesis.

## 2. Mechanisms and Pathophysiology

IgAN is an autoimmune disease. A significant body of evidence confirms the pivotal role of the multi-hit hypothesis in IgAN pathogenesis [13]. Specifically, the overproduction of polymeric Gd-IgA1 (hit 1) is recognized as an autoantigen, which induces the generation of circulating antiglycan autoantibodies, such as IgG and IgA (hit 2). Subsequently, the circulation of immune complexes (Gd-IgA1-CIC) is mainly formed via the direct versus between Gd-IgA1 and IgG or IgA (hit 3) [14]. It seems likely that IgA1-containing immune complexes (IgA1-CICs) can also form through the intermolecular disulfides-mediated self-aggregation of polymeric IgA molecules, which are prone to kidney deposition [15]. Additionally, it was proposed that abnormal binding between circulating IgA1 and the soluble form of the myeloid IgA Fc α receptor CD89 induces the formation of IgA1–sCD89 complexes [16]. The complexes deposit within mesangial cells via mesangial trapping and the high affinity of Gd-IgA1 for extracellular matrix components, leading to glomerular injury (hit 4) [9]. IgA1/IgA-CICs in circulation or after their deposition in the mesangial area further activate complement pathways, leading to glomerular inflammation and tubulointerstitial scarring [10,11] (Table 1).

Noticeably, the multi-hit theory still cannot fully explain some clinical, pathological and epidemiological observations in patients with IgAN [11]. For example, roughly 5% of biopsy-proven IgAN patients presented minor glomerular injury and mild or even no damage to kidney function [4]. Conversely, up to 16% of healthy individuals showed deposited glomerular IgA without any signs of kidney disease [5]. Recent evidence from genetic, immunological and molecular bases provided us with a better understanding of the pathogenesis of IgAN.

### 2.1. Genetic Susceptibility

The incidence of IgAN is reported to have a clear west-to-east prevalence gradient, with the disease being most common in Asia (45–58% in China, 47% in Japan, 28% in Korea and 45% in Singapore) when compared with Europe (10–35%) [12]. Familial aggregation of IgAN was well-described throughout the world [17]. The geographic diversity in prevalence and the heritable trait of Gd-IgA1 emphasize the potential contributions of both environment and genetics to IgAN susceptibility. Genome-wide association studies (GWASs) have remarkably advanced our understanding of the pathogenesis of IgAN by identifying IgAN susceptibility loci. The results from recent large GWASs showed that most susceptibility loci are involved in IgAN pathogenesis-related immunologic and inflammatory pathways, including mucosal immune response (CARD9; 9q34.3 and HORMAD2; 22q12), Gd-IgA1 production (ST6GAL1; 3q27, C1GALT1; 7p22.1-p21.3, GALNT12; 9q22.33, FCRL3; 1q23.1 and TNFSF13; 17p13), innate and adaptive immunity (MHC; 6p21), and complement activation (CFHR1; 1q32 CFHR3; 22q12, CFB; 6p21.33 and ITGAM–ITGAX; 16p11.2) [18,19].

### 2.2. Mucosal Immunity

**Figure 1:**
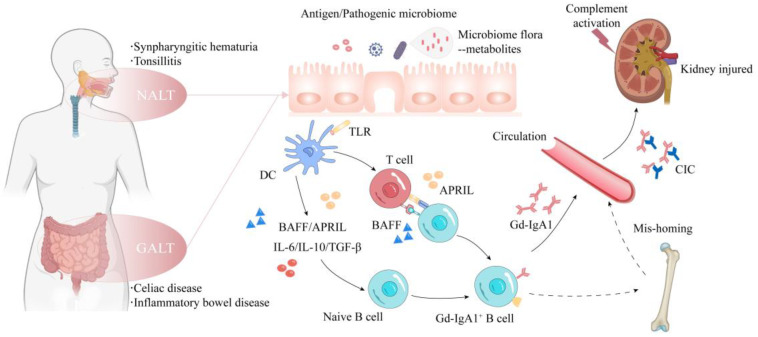
The pathophysiology of IgAN. IgAN usually presents with episodic gross hematuria and a concurrent upper respiratory tract infection or gastrointestinal disorder. Individuals with genetic susceptibility to IgAN develop aberrant mucosal immune responses to persistent antigenic stimulus, pathogenic microbiota and microbial metabolites. Antigen-presenting cells thereby activate and facilitate B-cells undergoing class switch and then differentiate into IgA+ ASCs in T-cell-dependent or T-cell-independent manners. Some mistrafficking ASCs take up residence in systemic sites, such as the bone marrow, NALT and GALT, leading to an increased circulating level of Gd-IgA1 and thus resulting in immune complex formation. The Gd-IgA1-containing immune complexes deposit in glomerular mesangium. Subsequently, complement activation (the alternative, lectin and terminal pathway) and intraglomerular inflammation together synergistically lead to kidney injuries, such as mesangial hypercellularity and fibrosis. ASC, antibody-secreting cell; CIC, circulating immune complex; DC, dendritic cell; NALT, nasal-associated lymphoid tissue; GALT, gut-associated lymphoid tissue; IgAN, IgA nephropathy.

Mucosal IgA production is an indispensable immunological process for host health. Clinically, the onset of mucosal infections, such as respiratory or gastrointestinal infections, often precipitates IgAN exacerbation, correlating IgAN with mucosal immunity [10]. Although the exact production site of Gd-IgA1 remains to be determined, considerable evidence supports that the mucosa-associated lymphoid tissue (MALT) is responsible for Gd-IgA1 production [20]. MALT is a key player in defending against environmental microbes and maintaining immunotolerance [21,22]. MALT distributes along the surfaces of all mucosal tissues, of which the two major regions related to IgAN are nasopharynx-associated lymphoid tissue (NALT) and gut-associated lymphoid tissue (GALT) [23,24]. In these inductive sites, following an encounter with mucosal-derived antigens or exogenous antigens, antigen-presenting cells (APC) activate naïve B-cells class-switching into IgA antibody-secreting cells (IgA+ ASC) via T-cell-dependent (TCD) and T-cell-independent (TCI) pathways (Figure 1). In the TCD manner, activated helper T-cells promote B-cell class switch by T-cell membrane-bound cytokine CD40 ligand and action of the cytokines, such as the transforming growth factor (TGF-β) 1, IL6 and IL10 [25]. TCI is induced by cytokines derived from the toll-like receptor (TLR) ligand-activated APCs, such as IL-6 and IL-10, TGFβ, as well as B-cell activating factor (BAFF) and a proliferation-inducing ligand (APRIL) [10,26]. Specifically, BAFF can be recognized by three different receptors (e.g., BAFF receptor (BAFF-R), transmembrane activator and CAML interactor (TACI) and B-cell maturation antigen (BCMA)) [25]. (a) BAFF can induce TCI class switching to generate both IgA and IgG antibodies [3]. (b) BAFF can also activate APRIL–TACI Axis for further generation of IgA [3]. (c) TACI can be coupled with myeloid differentiation factor 88 (MyD88) to induce B-cell class switching to produce IgA [25]. Persistent and excessive activation of TLRs might promote Gd-IgA1 production and downstream events [27]. Then, activated B-cells differentiate into IgA+ plasmablasts and are home to systemic effector sites. Intriguingly, B-cells derived from NALT and GALT exhibit different homing tendencies due to the varied combinations of chemokine receptors and adhesion molecules. B-cells derived from NALT tend to home to the mucosa and lymphoid tissue throughout the body, whereas the GALT-induced B-cells tend to home back to GALT [22].

IgA+ ASC in MALT can become memory long-lived plasma cells (LLPCs), producing long duration of IgA antibodies [10,28]. Regarding the origin of Gd-IgA1-CIC, it is presumed that the mucosal hyperresponsiveness of IgA1 might result in a ‘spillover’ of Gd-IgA1 into circulation. An alternative hypothesis was proposed, positing that some of these LLPCs or activated mucosal B-cells may mis-migrate into the bone marrow or other systemic sites instead of homing to mucosa sites. The inappropriate expression of surface homing receptors on lymphocyte subsets or the faulty expression of mucosal chemokines and homing counter-receptors on mucosal vascular endothelium may explain the postulation [20,29].

### 2.3. NALT

Human NALT consists mainly of Waldeyer’s pharyngeal ring, which is constituted by the adenoids and the lingual tonsils, the palatine tonsils and the eustachian tonsils. It is supposed that tonsillar NALT may be related to the onset and progression of IgAN [30,31]. Moreover, tonsillectomy aims to eliminate the source of mucosa-associated pathogens. This was shown to arrest or reduce serum level and mesangial deposition of Gd-IgA1, although the effectiveness of tonsillectomy therapy is clinically variable [32,33,34]. Despite being different from human NALT structures [35], the findings from murine IgAN models also showed that NALT structures located in the floor of the nasal cavity were the major induction site of aberrant Gd-IgA1 and nephritogenic ICs [23].

The robust findings from recent studies have indicated the relationship between specific microbial stimulation and NALT immunity in IgAN [36]. For example, the Sendai virus, or respiratory syncytial virus, was found to promote mesangial cells to release IL-6 and prostaglandin E2, which in turn induced mesangial proliferation [30,31]. The oral indigenous bacterium, Haemophilus parainfluenzae, was indicated to facilitate tonsillar mononuclear cell proliferation and promote IgA production in vitro [37]. The collagen-binding protein-positive Streptococcus mutans strains, which are isolated from the oral cavity, have shown their association with immunofluorescent Gd-IgA1 staining intensity and the clinical features of IgAN [38]. Comprehensive microbiome analysis revealed that Prevotella spp., Fusobacterium spp., Sphingomonas spp. and Treponema spp. were the predominant bacteria in both IgAN and recurrent tonsillitis [39]. The relative abundances of Rahnella, Ruminococcus_g2 and Clostridium_g21 in IgAN tonsils were shown to be different from those of healthy controls and other GN controls [36].

### 2.4. GALT

The GALT origin of Gd-IgA1-secreting cells was elucidated by Sallustio and his colleagues [40]. Zachova et al. consistently found that increased circulating B-cells express surface Gd-IgA1 and chemokine receptor CCR10, which are predestined for homing to the gut. All these findings indicate the role of GALT dysregulation in IgAN pathogenesis [35].

The gastrointestinal microbiota exists in reciprocal balance with the GALT. The imbalance of the gastrointestinal flora, namely gut dysbiosis, leads to the compromised intestinal epithelial barrier function and impaired immune response in patients with IgAN [41]. The immune response is clinically associated with inflammatory bowel disease (IBD) and celiac disease [42]. Gut dysbiosis is caused by changes in the structure and composition of the normal gut microbiome [41]. Several fecal microbiota investigations have shown the differences in microbial diversity between IgAN patients and healthy individuals. At phylum/genera/species levels, Sutterellaceae and Enterobacteriaceae species, Escherichia–Shigella, Defluviitaleaceae_incertae_sedis, Firmicutes, Ruminococcaceae, Lachnospiraceae, Eubacteriaceae and Streptococcaeae showed higher abundance in the stool samples of IgAN patients. At the same time, Bifidobacterium species, Roseburia, Lachnospiraceae_unclassified, Clostridium_sensu_stricto_1, Haemophilus and Fusobacterium showed an opposite trend [43,44]. Furthermore, several studies identified the associations between the composition of commensal microbiota and IgA production. Increases in Bacteroides spp, Lactobacillus spp, Clostridium coccoides, Bacteroides ovatus and Alcaligenes and decreases in Bifidobacterium spp. were demonstrated to strongly stimulate the production of IgA in mice [45,46,47,48].

Not only gut microbiota itself but also its metabolites participate in the GALT immune responses in IgAN. For example, lipopolysaccharide (LPS) and lipoteichoic acid, which are the ligands for TLR4 and TLR2 separately, may activate GALT and lead to the defective galactosylation of IgA1 [49]. Gut-derived uremic toxins, such as indoxyl sulfate, p-cresyl sulfate, indole-3 acetic acid, trimethylamine N-oxide and phenylacetylglutamine, were also found to induce intestinal inflammation and IgA overproduction via the increased release of cytokines (TNF-α, IFN-γ, IL-1β, IL-12) [50,51]. Sallustio et al. found that the amount of five specific gut microbiota metabolites (p-tert-butyl-phenol, 4-(1,1,3,3-tetramethylbutyl) phenol, hexadecyl ester benzoic acid, methyl neopentyl phthalic acid and furanone A) positively related to the serum levels of BAFF, among which phenol can increase gut permeability and lead to mucosal hyper-responsivity in IgAN patients [40]. On the contrary, there are also some vital metabolites whose decrease might intensify the progression of the disease. Short-chain fatty acids (SCFAs) are essential in gut barrier protection and the host immune system [52]. Bile acids (BAs) could prevent the host from inflammatory colitis by alleviating its susceptibility [53]. Therefore, the decrease of SCFAs and BAs caused by gut dysbiosis might increase the production of IgA [54].

The emerging evidence confirms the synergetic role of diet in augmenting IgA/Gd-IgA1 generation. For example, a high-fat diet (HFD) has been linked to IgAN by decreasing IgA-producing plasma cells, inducing inflammation and gut microbiota dysbiosis in mice colonic lamina propria [55,56]. Gluto was shown to promote the IgA1 production and the IgA1–sCD89 ICs’ formation in an IgAN mouse model [57]. There is also evidence that gluten-free diets may reduce mesangial IgA1 deposits and hematuria [57]. Additionally, the synthesis of IgA-CICs in patients with IgAN was also shown to be boosted by two other dietary antigens, namely bovine serum albumin and β-lactoglobulin [58,59]. These findings have provided a view of microbiota in the regulation of MALT immune responses in the development of IgAN.

**Table 1:** Molecules involved in the pathogenesis of IgAN.

Mechanism	Subjects	Pathophysiologic Processes	Reference
Multi-hit Hypothesis	Gd-IgA1	Formation of pathogenic circulating immune complexes	[13]
	Anti-Gd-IgA1 antibodies		[14]
	Soluble CD89		[16]
Mucosal Immunity	CD40	Promoting B-cell class switch	[10,25,26]
	BAFF/APRIL		
	Transforming growth factor β, Interleukin 6, Interleukin 10		
	TLRs	Activating the innate immune responses	
	Gut-derived uremic toxins (Indoxyl sulfate, p-cresyl sulfate, indole-3 acetic acid, trimethylamine N-oxide and phenylacetylglutamine)	Inducing intestinal inflammation and IgA overproduction	[50,51]
	Gut microbiota metabolites (p-tert-butyl-phenol, 4-(1,1,3,3-tetramethylbutyl) phenol, hexadecyl ester benzoic acid, methyl neopentyl phthalic acid and furanone A)	Increasing gut permeability and inducing mucosal hyper-responsivity	[40]
	Short-chain fatty acids	Maintaining gut barrier function and host immune system	[52,54]
	Bile acids	Regulation of intestinal immune responses	[53,54]
Complement Pathways	C3	Activation of alternative pathway	[60]
	Alternative pathway proteins (Complement factor H-related protein 1 and 5)	Competitively inhibiting factor H and leading to overactivation of alternative pathway	[61,62]
	Lectin pathway proteins (MBL, L-ficolin, M-ficolin, MASP 1/3 and MAp19)	Promoting activation of lectin pathway	[63,64,65]

Galactose-deficient IgA1 (Gd-IgA1); B-cell activating factor (BAFF); A proliferation-inducing ligand (APRIL); Toll-like receptors (TLRs).

### 2.5. Complement Dysregulation

Recent studies confirmed that the complement alternative pathway (AP), lectin pathway (LP) and terminal pathway (TP) are crucial contributors to kidney damage in IgAN [11]. It was proven that C3 deposition, which exists in more than 90% of IgAN biopsies, is related to AP overactivity [60]. Complement factor H (FH) is a key regulator of AP activation and amplification. The FH-related (FHR) proteins have a similar structure to FH but lack the complement regulatory FH region. AP proteins, such as properdin, FH, FHR1 and FHR5 are associated with progressive IgAN. Concomitantly, low C3 and high iC3b, as well as C3d in plasma, might correlate with worse kidney outcomes. Plasma levels of FHR1 and FHR5 are higher in IgAN patients, which might be related to disease progression [61,62]. The glomerular deposition of specific LP proteins, such as mannan-binding lectin (MBL), L-ficolin, MBL-associated serine proteases (MASP)1/3 and C4d deposition are also involved in IgAN progression [63,64]. In plasma, decreased levels of MASP-3 and increased levels of L-ficolin, M-ficolin, MASP-1 and MAp19 are correlated with IgAN severity [65]. The deposition of TP component C5b9 induces mesangial cell apoptosis and glomerular inflammation via IL-6 and TGF-β1 production, ultimately leading to glomerulosclerosis [66]. Additionally, a genetic analysis also found that an allele within the FH gene (delCFHR3-R1) could reduce mesangial C3 deposition and kidney injury by enhancing the complement AP regulation [64]. MBL2 variant rs1800450-A, which is related to reduced MBL plasma levels, can influence susceptibility to severe tubulointerstitial damage [67].

## 3. Prediction and Prognosis of IgAN

Proteinuria is consistently demonstrated to be a dominant risk factor for disease progression in IgAN [68]. The presence and the amount of proteinuria are closely associated with declined kidney function. In the 2021 Kidney Disease Improving Global Outcomes (KDIGO) glomerulonephritis guidelines [69], a high risk of progression in IgAN is defined as proteinuria > 0.75–1 g/d despite 90 days of optimized supportive care, and a reduction to under 1 g/d is a surrogate marker of improved kidney outcome.

A diagnostic kidney biopsy is mandatory and provides important prognostic information for IgAN. The Oxford MEST-C scoring system offered the first opportunity to use histology to predict kidney outcomes independent of the clinical parameters at the time of biopsy [70,71,72]. The scoring system includes five histologic features: mesangial (M) and endocapillary (E) hypercellularity, segmental sclerosis (S), interstitial fibrosis/tubular atrophy (T) and Crescent(C). The VALIGA study confirmed the association of M1, S1 and T1/2 with kidney outcomes and the association of M1 and E1 with subsequent increases in proteinuria in IgAN [5]. Combining clinical markers and the MEST-C score may be a better predictor of kidney survival in IgAN. Until recently, as endorsed by the 2021 KDIGO guidelines, it is capable of predicting up to 6.7 years ahead of the risk of a 50% decrease in eGFR or the development of kidney failure with the newly developed International IgAN Prediction Tool in 2019 [73]. However, the tool may only be applied at the time of the kidney biopsy. Based on that, the research group refined the Prediction Tool by enrolling 2507 IgAN adults in an international multi-ethnic cohort study [74]. Noticeably, the cohort included IgAN patients with a wide range of proteinuria since many of them would have been considered low-risk based on proteinuria alone. Not only does it have similar prediction performance, but the updated Prediction Tool can also be used two years after the biopsy.

The current clinical markers and kidney biopsy findings of IgAN, namely proteinuria, hypertension, eGFR and the MEST-C score, are non-specific. New biomarkers are needed to improve the prognosis of IgAN. Although no validated prognostic biomarkers in serum or urine can be adopted into clinical practice, there are over 1000 derivation studies taking a leap forward (Table 2).

The multi-hit hypothesis is the main direction in this field. Take serum Gd-IgA1 as an example; the levels of Gd-IgA1 are significantly higher in patients with IgAN than in healthy controls or patients with other kidney diseases [75,76,77,78]. Furthermore, Zhao et al. demonstrated significant associations between higher serum Gd-IgA1 levels and a greater risk of progression in IgAN [79]. In the secondary hit, the outcomes in the French IgAN cohort study showed that the serum levels of Gd-IgA1-specific IgG and IgA autoantibodies were associated with the risk of disease progression [80]. In the third hit, Zhang et al. confirmed that a higher level of recombinant CD89-bound poly-IgA immune complex was associated with the severity of the disease and with treatment response to steroids and immunosuppressants [81]. The complement activation was shown to occur on hit three and hit four, initiating and amplifying glomerular injury. Histopathology for complement proteins could help to determine disease activity and severity. Glomerular C4d deposition may represent its potential to be a marker for adverse prognosis [63]. As a complement factor in the lectin pathway, C4d deposition was found to contribute to pathogenic mechanisms in IgAN and is correlated with eGFR decline, proteinuria and severe kidney damage [82].

Urine, a noninvasive, low proteolytic active body fluid, is a promising field for developing potential biomarkers [83]. Normal human urine contains at least 5000 naturally occurring peptides originating from glomerular filtration, tubular secretion and epithelial cells. It reflects the physiological and pathophysiological processes in the body. Urinary laminin G-like 3 and free kappa light chains are inversely correlated with the severity of clinical features and prognosis of IgAN [84]. Urinary Dickkopf-3, a stress-induced tubular epithelial-derived profibrotic glycoprotein, is promising to predict the course of kidney function over the next 12 months [85].

MicroRNAs (miRNAs) are short and noncoding oligonucleotides. It can regulate gene expression by disrupting translation, which has also shown values in IgAN. The first research on miRNAs in IgAN described the relationship between the urinary sediment miR-200 family, miR-205 and miR-192 and the severity of kidney injury in IgAN [86]. Moreover, Serino et al. found that miR-148b was upregulated in peripheral blood mononuclear cells from IgAN patients, resulting in a reduction of C1GALT1. The level of miR-148b positively correlated with the serum level of Gd-IgA1 in IgAN patients [87]. Moreover, isolated peripheral B-cells transfected with miR-374b had significantly increased the expression of Cosmc, a galactosyltransferase involved in Gd-IgA1 production [88]. The level of miR-374b in B-cells was positively related to urine protein level and pathological MEST score.

**Table 2:** Tools and biomarkers in the prediction and prognosis of IgAN.

	Subjects	Origin	Clinical Significance	Reference
Tools	MEST-C score	Histopathology	Histopathological lesions associated with adverse outcomes	[5,70,71,72]
	The International IgAN Prediction Tool	Clinical and histopathological parameters	Quantifying the risk of progression up to 6.7 years after biopsy	[73,74]
Biomarkers	Proteinuria	Urine	Risk factor for disease progression of IgAN	[68]
	Serum Gd-IgA1	Serum	Independent risk factor for disease progression of IgAN	[79]
	Gd-IgA1-specific IgG and IgA autoantibodies	Serum	Association with the risk of disease progression	[80]
	Recombinant CD89-bound poly-IgA immune complex	Serum	Association with the severity of IgAN and with treatment response to steroids and immunosuppressants	[81]
	C4d	Histopathology	Association with clinical and histopathological severity of IgAN	[63,82]
	Laminin G-like 3	Urine	Association with clinical severity and prognosis	[84]
	Free kappa light chains	Urine		
	Urinary Dickkopf-3	Urine	Predicting kidney prognosis over the next 12 months	[85]
	miR-200 family, miR-205, miR-192	Urine	Association with disease severity and rate of progression	[86]
	miR-148b	Peripheral blood mononuclear cell	The level of miR-148b positively correlated with serum level of Gd-IgA1 in IgAN patients	[87]
	miR-374b	Blood B-cell	The level of miR-374b in B-cells was positively related to urine protein level and pathological MEST score	[88]

## 4. Current Treatment of IgAN

To date, there is no disease-specific therapy available for IgAN since the pathogenesis mechanisms of IgAN are incompletely determined. The overall therapeutic aim of IgAN patients is to retard disease development and progression, which is generally achieved through optimal supportive care. In-depth insights into pathophysiology allow novel therapeutic approaches to provide treatment options for patients suffering from IgAN (Table 3).

### 4.1. Supportive Therapy

An optimal and comprehensive supportive treatment regimen was suggested by the KDIGO guidelines for IgAN patients, except for those with nephrotic syndrome or rapidly progressive IgAN [69,89]. More specifically, it includes antihypertensive and antiproteinuric medications, intensive education for lifestyle changes and avoidance of nephrotoxicity combinations. Currently, renin-angiotensin system (RAS) blockade is still the first-line treatment for IgAN patients due to its efficacy in reducing proteinuria and retarding disease progression [90]. However, the results from the STOP-IgAN subcohort study showed that a single RAS blockade (RASB) strategy exhibited antiproteinuric efficacy without affecting outcomes like full clinical remission, eGFR drop rates and kidney failure occurrence [91]. Several emerging supportive approaches further expand the armamentarium of the single RASB therapy. Sparsentan is a dual endothelin angiotensin receptor antagonist (DEARA). Its reno-protective potential is currently being evaluated in patients with high-risk IgAN in the phase III PROTECT trial (NCT03762850). The selective endothelin A receptor inhibitor, atrasentan, is also being tested in an ongoing phase III ALIGN study (NCT04573478). In addition, sodium-glucose cotransporter-2 inhibitors (SGLT2i) emerge as a promising novel approach in the treatment of IgAN. Results of a pre-specified analysis from the DAPA-CKD trial showed that adding dapagliflozin on top of RASB slowed kidney function decline versus placebo in IgAN patients [92]. Although the mechanisms by which dapagliflozin protect kidney function and slow down further progression are not fully understood, the early evidence demonstrated its remarkable kidney benefits and safety profiles in treating IgAN. Similarly, in the EMPA-KIDNEY study (NCT03594110), 817 IgAN patients were treated with long-term empagliflozin in addition to optimized supportive care. The outcomes are awaited.

### 4.2. Immunosuppression Therapy

The KDIGO guidelines note that IgAN patients who remain at a high risk of disease progression despite at least a 90-day course of optimized supportive care should consider a six months course of systemic corticosteroid therapy. However, the efficacy and safety of corticosteroids remain controversial. The STOP-IgAN trial brought negative results of corticosteroid therapy in addition to optimized supportive care in kidney outcomes, neither at three years after the trial randomization nor at prolonged follow-ups of a median of more than seven years [2,93]. Moreover, reported adverse events were significantly increased in the corticosteroid arm, particularly infections. Although treatment with oral methylprednisolone reduced time-averaged mean proteinuria and eGFR-slope in high-risk IgAN patients, the TESTING study supported safety concerns addressed by STOP-IgAN and thereafter came to an early termination [94]. The follow-up study of the TESTING trial reduced the methylprednisolone dose by 50%, combined with a 12-week antibiotic prophylaxis for pneumocystis pneumonia [95]. After a median follow-up of 2.5 years, there was no significant difference in treatment efficacy between the low-dose and original full-dose corticosteroid regimen. In terms of the primary endpoint, proteinuria reduction and kidney function loss, oral corticosteroids showed overall reno-protective effects over placebo [96]. It is worth noting that a low-dose regimen had a lower risk of adverse events compared with a full-dose regimen. Given the above, none of these studies provided a definite answer for the long-term efficacy of corticosteroids since the renoprotection effects might decrease over time. However, it is clear that high-dose and long-term systemic corticosteroid therapy will increase the risk of infection or adverse events in IgAN patients.

The KDIGO guidelines also recommended several immunosuppressants or immunomodulators beyond corticosteroids in IgAN. Mycophenolate mofetil (MMF), a B-lympholytic agent, exhibited beneficial effects in proteinuria reduction and further renoprotection in Chinese IgAN patients [97,98]. However, the studies conducted on Caucasian patients produced conflicting data [99]. MMF was, therefore, recommended as a corticosteroid-sparing agent only in Chinese patients by KDIGO. Hydroxychloroquine (HCQ) is an antimalarial agent and also a TLR-9 inhibitor which is known to be involved in the production of Gd-IgA1 and inflammatory responses suppression of class-switched memory B-cells [100,101]. HCQ significantly decreased proteinuria in IgAN patients without evidence of adverse events [102,103]. Likewise, a retrospective study showed that long-term HCQ monotherapy could reduce proteinuria and retard kidney function deterioration in IgAN patients with different eGFR [104]. However, the intervention timing of HCQ, whether as an additional initiative supportive therapy or a supplement for insufficient conventional immunosuppression therapy, is required to be confirmed [105].

### 4.3. Outlook of Future Alternatives to Conventional Immunosuppression

Mounting evidence supports that immunological events are engaging in every aspect of IgAN pathogenesis, especially in genetically susceptible individuals. General immunosuppression therapies, like systemic corticosteroids, would raise the risk of disruptive adaptive immunity and infection. In light of the updated pathophysiological insights into IgAN, novel and promising therapeutic approaches were designed to target different processes in IgAN. The adoption of these more targeted approaches would potentially improve the risk/benefit profile in patients with IgAN.

#### 4.3.1. Regulation of Pathogenic IgA1 and CIC Production

- Inhibition of TLRs/BAFF/APRIL signaling

An increasing number of studies revealed that LLPCs might be responsible for the production of Gd-IgA1 and anti-Gd-IgA1 antibodies. The activation of TLRs and an up-regulation of BAFF/APRIL signaling are significantly related to the function and survival of B-cell and plasma cells [100,106,107,108].

As mentioned above, HCQ inhibited excessive TLR activation and has shown its potential in renoprotection and anti-inflammation in IgAN patients [102].

Blisibimod is a monoclonal antibody against both soluble and membrane BAFF. As shown by the interim results of the phase II/III BRIGHT-SC study (NCT02062684) in Kidney Week of the American Society of Nephrology 2016, blisibimod-mediated BAFF inhibition can reduce the level of peripheral B-cells, immunoglobulins and urine protein-to-creatinine ratio (UPCR) when compared with placebo in patients with IgAN.

Anti-APRIL antibodies are also under evaluation in clinical studies. VIS649 is a humanized IgG2κ antibody, which has been tested for its therapeutic efficacy and safety profile in preclinical research and in a phase I study in healthy subjects (NCT03719443) [109,110]. Similarly, humanized IgG4 anti-APRIL monoclonal antibody BION-1301 is being assessed by phase I/II clinical trials (NCT03945318).

Atacicept, a soluble TACI-Immunoglobin fusion protein, may dually inhibit BAFF- and APRIL-mediated B-cell class switching to reduce antibody levels. The phase II JANUS study (NCT02808429) demonstrated the potential of atacicept in the renoprotection and Gd-IgA1 reduction in high-risk IgAN patients [111]. A larger clinical trial (the ORIGIN phase IIb study, NCT04716231) further assesses its tolerability and safety, which is now undergoing. Likewise, another ongoing phase II trial was conducted to determine the safety and efficacy of Telitacicept, which is a BAFF/APRIL dual inhibitor (NCT04291781 and NCT04905212).

- Depletion of Gd-IgA1-producing plasma cells

Anti-CD20 antibody therapeutics aim to delete B-cells in the long-term treatment of IgAN. Like rituximab, it was demonstrated to deplete B-cells effectively but failed to eliminate LLPCs and the serum levels of Gd-IgA1 and Gd-IgA1 antibodies [112,113]. Additionally, strategies targeting CD38+ plasma cells hold great promise in future applications. A phase IIa clinical trial (IGNAZ study, NCT05065970) of felzartamab, a fully human IgG1 monoclonal antibody designed to deplete CD38+ plasma cells, is currently underway in IgAN patients at high risk of progression.

#### 4.3.2. Clearance of IgA Deposits

Regarding the excessive pathogenic IgA1 and CICs, enhancing the functions of IgA receptors and developing potent IgA1 proteases to dissolve away mesangial IgA deposits offer a plausible proposition for the treatment of IgAN. The FcαRI (CD89) expressed on blood myeloid cells and the transferrin receptor (CD71) expressed on mesangial cells are two important IgA receptors. They both account for the initiation, progression and chronicity of IgAN [114,115,116]. The bacteria-derived IgA proteases were found to be effective in the cleavage of IgA1 and IgA1-containing ICs. Moreover, IgA proteases could diminish mesangial deposition of IgA complexes [117,118] and reduce circulating IgA1 and IgA1–sCD89 complexes in the α1KI-CD89Tg mouse model of IgAN [119]. Recently, Xie et al. constructed a recombinant fusion protein termed Fc-AK183, which was fused by IgA protease from a commensal bacteria, Clostridium Ramosum, and human IgG Fc. Fc-AK183 provided long-lasting clearance of both circulating and mesangial deposited IgA as well as C3 in IgAN rodent models [120]. Nonetheless, these preliminary findings are still waiting for translation into clinical utilities. Fostamatinib, an oral prodrug spleen tyrosine kinase inhibitor, was investigated by a phase II SIGN study (NCT02112838) [121]. It might also be a novel treatment by targeting autoantibody production and downstream inflammatory response in mesangium [122,123].

#### 4.3.3. Modulation of Mucosal Immunity

- Modulation of NALT

Evidence suggested that NALT or palatine tonsils may also be responsible for the synthesis of Gd-IgA1 in patients with IgAN [23,33,124]. Although several studies show that tonsillectomy could reduce the serum levels of Gd-IgA1 [125,126], data from European and Asian studies revealed that tonsillectomy is associated with an increased likelihood of developing IBD and irritable bowel syndrome [127,128]. Thus, novel treatment strategies targeting NALT are still needed. A pilot study of a topical steroid gargle targeting NALT for patients with IgAN is now recruiting to assess its efficacy and safety (Preprint) [129].

- Modulation of GALT and Gut microbiota
  1. **Gut-targeted immunosuppression**
    Evidence suggests that the GALT, including Peyer’s patches, may play a critical role as a potential source of Gd-IgA1 in IgAN, where antigens, microbes or products of gut microbial activity initiate mucosal pathogenic IgA synthesis [130]. Hence, immunosuppression targeted to dysregulated GALT immune responses may provide an alternative to conventional systemic immunosuppression. Tarpeyo is a distal ileum targeted-release budesonide, which has been shown to reduce the level of CICs in a dose-dependent manner [131]. Recently, tarpeyo was granted accelerated approval by the FDA since two clinical trials (NEFIGAN, NCT01738035; NefIgArd Part A, NCT03643965) preliminarily demonstrated its efficacy in reducing proteinuria and stabilizing kidney function despite optimized RAS inhibition [132]. Zhang et al. developed a novel ileocecum targeting medication based on an orange-derived and dexamethasone-encapsulated extracellular vesicle (EVs-DexP) delivery system [133]. Evs-DexP exerted its effects by reducing intestinal IgA production and kidney IgA deposition in IgAN mice. It can also suppress lymphocyte activation in vitro while decreasing the ratio of IgA+B220+ lymphocytes in Peyer’s patches.
  2. **Gut microbiota modulation**
    Several studies indicated that dysbiosis of the gut microbiota might be associated with the progression, clinical features and treatment responses of IgAN [134,135,136]. Coupled with the pathogenesis of IgAN, approaches focused on the restoration of intestinal flora homeostasis, such as regulation of microbial diversity and metabolites, would be promising adjuvant therapeutic options against IgAN. Broad-spectrum antibiotics exhibited therapeutic effects on modulating microbiota, resulting in reduced IgA1-related CICs and mesangial IgA1 deposition in humanized mouse models of IgAN [137,138]. Another potential option is regulating gut immunity by the supplementation of probiotics/prebiotics/specific microbial metabolites or by the transplantation of fecal microbiota [139,140,141]. Some of these strategies have been shown to improve pathophysiological and clinical parameters in IgAN patients.

#### 4.3.4. Blockade of Complement Cascades

Several ongoing phase II/III trials have targeted downstream factors of C5. For instance, CCX168, an anti-C5a receptor antagonist, showed an improvement in the slope of the UPCR in a short-term pilot study (NCT02384317) [142]. ALXN1210, as an eculizumab-derived long-acting C5-blocking antibody (SANCTUARY study, NCT04564339), and ALN-CC5, as a small interfering RNA-targeting C5 (NCT03841448), are being evaluated for their safety, tolerability and efficacy in IgAN patients with results awaited. While other complement-directed therapies targeting AP or LP are also pursued as promising options for IgAN in their underway phase II/III trials. APL-2, an AP inhibitor preventing C3 activation, is being tested in a phase II trial for preliminary efficacy (NCT03453619). LNP023 is a promising oral selective factor B inhibitor. It provides sustained inhibition of AP activity while exhibiting a continuous antiproteinuric effect and a well-tolerated safety profile in a phase II trial (NCT03373461) [143]. Phase III APPLAUSE-IgAN trial of LNP023 (NCT04578834) is underway based on these results. Moreover, an anticomplement factor B monoclonal inhibitor, IONIS-FB-LRx, is currently being evaluated by a phase II trial (NCT04014335). Interim analysis of the anti-MASP-2 monoclonal antibody, OMS721, has indicated its potential benefits from the data of a staged phase II study (NCT02682407) [144]. The safety and efficacy of OMS721 in IgAN patients with more than 1 g/d proteinuria are currently being assessed in the phase III ARTEMIS-IGAN study (NCT03608033).

**Table 3:** Clinical trials assessing targeted therapies in IgAN.

Agent	Mechanism of Action	Registration No.	Phase	Primary Outcome Measures	Trial Results/Status	Reference
Hydroxychloroquine	TLR signaling inhibitor	NCT02942381	2	Proteinuria (every 2 months, total 6 months)	In addition to optimized renin-angiotensin–aldosterone system inhibition, hydroxychloroquine significantly reduced proteinuria without evidence of adverse event	[102]
Blisibimod	Monoclonal antibody of soluble and membrane BAFF	NCT02062684	2/3	Proportion of subjects achieving reduction in proteinuria from baseline (24 weeks)	The interim results of blisibimod treatment showed a reduction in the level of proteinuria, peripheral B-cells and immunoglobulins	-
VIS649	Monoclonal IgG2κ antibody targeting APRIL	NCT03719443	1	Number of participants with adverse events and frequency of ECG abnormalities (112 days)	VIS649 treatment reduced serum levels of APRIL, IgA and Gd-IgA1 without evidence of severe adverse event	[109,110]
BION-1301	Monoclonal IgG4 antibody targeting APRIL	NCT03945318	1/2	Incidence and severity of adverse events (76 weeks)	Recruiting	-
Atacicept	BAFF/APRIL dual inhibitor	NCT02808429	2	Percentage of adverse events (96 weeks)	1. Atacicept treatment demonstrated an acceptable safety profile 2. The interim results of atacicept treatment showed early reduction in proteinuria and dose-dependent reduction in Gd-IgA1	[111]
		NCT04716231	2	Change from baseline in UPCR (24 weeks)	Active, not recruiting	-
Telitacicept	BAFF/APRIL dual inhibitor	NCT04291781	2	Change from baseline in 24-h urine protein excretion level (24 weeks)	Results not yet available	-
		NCT04905212	2		Recruiting	-
Rituximab	Monoclonal anti-CD20 antibody	NCT00498368	4	Change in proteinuria (12 months)	Rituximab treatment did not significantly improve kidney function or proteinuria and failed to reduce serum levels of Gd-IgA1 and anti-Gd-IgA1 antibodies	[113]
Felzartamab	Monoclonal IgG1 antibody targeting CD38	NCT05065970	2	Relative change in proteinuria value (9 months)	Recruiting	-
Fostamatinib	Spleen tyrosine kinase inhibitor	NCT02112838	2	Mean change from baseline in proteinuria (24 weeks)	Fostamatinib treatment did not significantly improve proteinuria or eGFR	[121]
NEFECON (TARPEYO)	Distal ileum targeted-release budesonide formulation targeting B-cells in mucosal lymphoid tissue	NCT01738035	2	Change from baseline in UPCR (9 months)	Nefecon treatment reduced proteinuria and preserved kidney function	[132]
		NCT03643965	3	Change in proteinuria (9 months) and eGFR (up to 2 years)		[131]
CCX168	Anti-C5a receptor antagonist	NCT02384317	2	The number of patients with adverse events (169 days)	CCX168 treatment improved proteinuria	[142]
ALXN1210	Long-acting C5-blocking antibody	NCT04564339	2	Percentage change from baseline in proteinuria (26 weeks)	Recruiting	-
ALN-CC5	Small interfering RNA targeting C5	NCT03841448	2	Percentage change from baseline in UPCR (32 weeks)	Active, not recruiting	-
APL-2	Cyclic peptide inhibitor of C3	NCT03453619	2	Proteinuria (48 weeks)	Active, not recruiting	-
LNP023	Selective complement factor B inhibitor	NCT03373461	2	Multiple comparison procedure modeling estimates of the ratio to baseline of UPCR (90 days)	LNP023 treatment led to continuous reduction in proteinuria and strong inhibition of alternative pathway activity	[143]
		NCT04578834	3	Ratio to baseline in UPCR (9 months) and annualized total eGFR slope (24 months)	Recruiting	-
IONIS-FB-LRx	Antisense inhibitor of complement factor B	NCT04014335	2	Percent reduction in 24-h urine protein excretion (29 weeks)	Recruiting	-
OMS721	Monoclonal antibody against mannan-associated lectin-binding serine protease-2	NCT02682407	2	Proportion of adverse events (Up to 104 weeks) and change from baseline in serum and urine complement component levels (38 weeks)	OMS721 treatment reduced proteinuria and preserved kidney function	[144]
		NCT03608033	3	Change from baseline in 24-h urine protein excretion (36 weeks)	Recruiting	-

UPCR: urine protein to creatinine ratio.

## 5. Conclusions

IgAN is an autoimmune disease arising from interactions of genetic, ethnic, environmental and nutritional contributing factors. It is characterized by vast heterogeneity in clinical presentation and prognosis between individuals. As discussed in this review, disease-oriented specific biomarkers with prognostic values and putative therapeutic approaches targeting updated pathogenesis processes are evaluated in ongoing clinical trials worldwide. Although tremendous progress in the understanding of the disease has been made, there are still a number of questions that require clarification in the coming years.

## Data Availability

Not applicable.

## References

[1] Lai K.N., Tang S.C., Schena F.P., Novak J., Tomino Y., Fogo A.B., Glassock R.J. (2016). IgA nephropathy. Nat. Rev. Dis. Prim..

[2] Rauen T., Wied S., Fitzner C., Eitner F., Sommerer C., Zeier M., Otte B., Panzer U., Budde K., Benck U. (2020). After ten years of follow-up, no difference between supportive care plus immunosuppression and supportive care alone in IgA nephropathy. Kidney Int..

[3] Yeo S.C., Cheung C.K., Barratt J. (2017). New insights into the pathogenesis of IgA nephropathy. Pediatr. Nephrol..

[4] D’Amico G. (2004). Natural history of idiopathic IgA nephropathy and factors predictive of disease outcome. Semin. Nephrol..

[5] Suzuki K., Honda K., Tanabe K., Toma H., Nihei H., Yamaguchi Y. (2003). Incidence of latent mesangial IgA deposition in renal allograft donors in Japan. Kidney Int..

[6] Gharavi A.G., Moldoveanu Z., Wyatt R., Barker C.V., Woodford S.Y., Lifton R.P., Mestecky J., Novak J., Julian B.A. (2008). Aberrant IgA1 Glycosylation Is Inherited in Familial and Sporadic IgA Nephropathy. J. Am. Soc. Nephrol..

[7] Nakazawa S., Imamura R., Kawamura M., Kato T., Abe T., Namba T., Iwatani H., Yamanaka K., Uemura M., Kishikawa H. (2018). Difference in IgA1 O-glycosylation between IgA deposition donors and IgA nephropathy recipients. Biochem. Biophys. Res. Commun..

[8] Gaber L.W., Khan F.N., Graviss E.A., Nguyen D.T., Moore L.W., Truong L.D., Barrios R.J., Suki W.N. (2020). Prevalence, Characteristics, and Outcomes of Incidental IgA Glomerular Deposits in Donor Kidneys. Kidney Int. Rep..

[9] Gesualdo L., Di Leo V., Coppo R. (2021). The mucosal immune system and IgA nephropathy. Semin. Immunopathol..

[10] He J.-W., Zhou X.-J., Lv J.-C., Zhang H. (2020). Perspectives on how mucosal immune responses, infections and gut microbiome shape IgA nephropathy and future therapies. Theranostics.

[11] Medjeral-Thomas N.R., Cook H.T., Pickering M.C. (2021). Complement activation in IgA nephropathy. Semin. Immunopathol..

[12] Schena F.P., Nistor I. (2018). Epidemiology of IgA Nephropathy: A Global Perspective. Semin. Nephrol..

[13] Suzuki H., Kiryluk K., Novak J., Moldoveanu Z., Herr A.B., Renfrow M.B., Wyatt R.J., Scolari F., Mestecky J., Gharavi A.G. (2011). The Pathophysiology of IgA Nephropathy. J. Am. Soc. Nephrol..

[14] Suzuki H. (2018). Biomarkers for IgA nephropathy on the basis of multi-hit pathogenesis. Clin. Exp. Nephrol..

[15] Xie X., Gao L., Liu P., Lv J., Lu W.-H., Zhang H., Jin J. (2021). Propensity of IgA to self-aggregate via tailpiece cysteine-471 and treatment of IgA nephropathy using cysteamine. J. Clin. Investig..

[16] Monteiro R.C. (2018). Recent advances in the physiopathology of IgA nephropathy. Néphrol. Thér..

[17] Kiryluk K., Li Y., Sanna-Cherchi S., Rohanizadegan M., Suzuki H., Eitner F., Snyder H.J., Choi M., Hou P., Scolari F. (2012). Geographic Differences in Genetic Susceptibility to IgA Nephropathy: GWAS Replication Study and Geospatial Risk Analysis. PLOS Genet..

[18] Ding X., Mei Y., Mao Z., Long L., Han Q., You Y., Zhu H. (2021). Association of Immune and Inflammatory Gene Polymorphism With the Risk of IgA Nephropathy: A Systematic Review and Meta-Analysis of 45 Studies. Front. Immunol..

[19] Zhou X.-J., Tsoi L.C., Hu Y., Patrick M.T., He K., Berthier C.C., Li Y., Wang Y.-N., Qi Y.-Y., Zhang Y.-M. (2021). Exome Chip Analyses and Genetic Risk for IgA Nephropathy among Han Chinese. Clin. J. Am. Soc. Nephrol..

[20] Knoppova B., Reily C., Maillard N., Rizk D.V., Moldoveanu Z., Mestecky J., Raska M., Renfrow M.B., Julian B.A., Novak J. (2016). The Origin and Activities of IgA1-Containing Immune Complexes in IgA Nephropathy. Front. Immunol..

[21] Gormley P.D., Powell-Richards A.O., Azuara-Blanco A., Donoso L.A., Dua H.S. (1998). Lymphocyte subsets in conjunctival mucosa-associated-lymphoid-tissue after exposure to retinal-S-antigen. Int. Ophthalmol..

[22] Kiyono H., Fukuyama S. (2004). NALT-versus Peyer’s-patch-mediated mucosal immunity. Nat. Rev. Immunol..

[23] Kano T., Suzuki H., Makita Y., Fukao Y., Suzuki Y. (2021). Nasal-associated lymphoid tissue is the major induction site for nephritogenic IgA in murine IgA nephropathy. Kidney Int..

[24] Barratt J., Rovin B.H., Cattran D., Floege J., Lafayette R., Tesar V., Trimarchi H., Zhang H., NefIgArd Study Steering Committee (2020). Why Target the Gut to Treat IgA Nephropathy?. Kidney Int. Rep..

[25] Cerutti A. (2008). The regulation of IgA class switching. Nat. Rev. Immunol..

[26] Nakawesi J., This S., Hütter J., Boucard-Jourdin M., Barateau V., Muleta K.G., Gooday L.J., Thomsen K.F., López A.G., Ulmert I. (2020). αvβ8 integrin-expression by BATF3-dependent dendritic cells facilitates early IgA responses to Rotavirus. Mucosal Immunol..

[27] Suzuki H., Suzuki Y., Narita I., Aizawa M., Kihara M., Yamanaka T., Kanou T., Tsukaguchi H., Novak J., Horikoshi S. (2008). Toll-Like Receptor 9 Affects Severity of IgA Nephropathy. J. Am. Soc. Nephrol..

[28] Lemke A., Kraft M., Roth K., Riedel R., Lammerding D., E Hauser A. (2016). Long-lived plasma cells are generated in mucosal immune responses and contribute to the bone marrow plasma cell pool in mice. Mucosal Immunol..

[29] Coppo R. (2018). Treatment of IgA nephropathy: Recent advances and prospects. Néphrol. Thér..

[30] Inoue T., Sugiyama H., Kitagawa M., Takiue K., Morinaga H., Kikumoto Y., Maeshima Y., Fukushima K., Nishizaki K., Akagi H. (2011). Abnormalities of Glycogenes in Tonsillar Lymphocytes in IgA Nephropathy. Adv. Otorhinolaryngol..

[31] Suzuki Y., Suzuki H., Nakata J., Sato D., Kajiyama T., Watanabe T., Tomino Y. (2011). Pathological Role of Tonsillar B Cells in IgA Nephropathy. J. Immunol. Res..

[32] Kawamura T., Yoshimura M., Miyazaki Y., Okamoto H., Kimura K., Hirano K., Matsushima M., Utsunomiya Y., Ogura M., Yokoo T. (2014). A multicenter randomized controlled trial of tonsillectomy combined with steroid pulse therapy in patients with immunoglobulin A nephropathy. Nephrol. Dial. Transplant..

[33] Kawabe M., Yamamoto I., Yamakawa T., Katsumata H., Isaka N., Katsuma A., Nakada Y., Kobayashi A., Koike K., Ueda H. (2020). Association Between Galactose-Deficient IgA1 Derived From the Tonsils and Recurrence of IgA Nephropathy in Patients Who Underwent Kidney Transplantation. Front. Immunol..

[34] Feehally J., Coppo R., Troyanov S., Bellur S.S., Cattran D., Cook T., Roberts I.S., Verhave J.C., Camilla R., Vergano L. (2015). Tonsillectomy in a European Cohort of 1,147 Patients with IgA Nephropathy. Nephron.

[35] Zachova K., Jemelkova J., Kosztyu P., Ohyama Y., Takahashi K., Zadrazil J., Orsag J., Matousovic K., Galuszkova D., Petejova N. (2022). Galactose-Deficient IgA1 B cells in the Circulation of IgA Nephropathy Patients Carry Preferentially Lambda Light Chains and Mucosal Homing Receptors. J. Am. Soc. Nephrol..

[36] Park J.I., Kim T.-Y., Oh B., Cho H., Kim J.E., Yoo S.H., Lee J.P., Kim Y.S., Chun J., Kim B.-S. (2020). Comparative analysis of the tonsillar microbiota in IgA nephropathy and other glomerular diseases. Sci. Rep..

[37] Fujieda S., Suzuki S., Sunaga H., Yamamoto H., Seki M., Sugimoto H., Saito H. (2000). Induction of IgA against Haemophilus parainfluenzae antigens in tonsillar mononuclear cells from patients with IgA nephropathy. Clin. Immunol..

[38] Ito S., Misaki T., Naka S., Wato K., Nagasawa Y., Nomura R., Otsugu M., Matsumoto-Nakano M., Nakano K., Kumagai H. (2019). Specific strains of Streptococcus mutans, a pathogen of dental caries, in the tonsils, are associated with IgA nephropathy. Sci. Rep..

[39] Watanabe H., Goto S., Mori H., Higashi K., Hosomichi K., Aizawa N., Takahashi N., Tsuchida M., Suzuki Y., Yamada T. (2016). Comprehensive microbiome analysis of tonsillar crypts in IgA nephropathy. Nephrol. Dial. Transplant..

[40] Nyangale E.P., Mottram D.S., Gibson G.R. (2012). Gut Microbial Activity, Implications for Health and Disease: The Potential Role of Metabolite Analysis. J. Proteome Res..

[41] Coppo R. (2014). The intestine-renal connection in IgA nephropathy. Nephrol. Dial. Transplant..

[42] Monteiro R.C., Rafeh D., Gleeson P.J. (2022). Is There a Role for Gut Microbiome Dysbiosis in IgA Nephropathy?. Microorganisms.

[43] De Angelis M., Montemurno E., Piccolo M., Vannini L., Lauriero G., Maranzano V., Gozzi G., Serrazanetti D., Dalfino G., Gobbetti M. (2014). Microbiota and metabolome associated with immunoglobulin A nephropathy (IgAN). PLoS ONE.

[44] Dong R., Bai M., Zhao J., Wang D., Ning X., Sun S. (2020). A Comparative Study of the Gut Microbiota Associated With Immunoglobulin a Nephropathy and Membranous Nephropathy. Front. Cell. Infect. Microbiol..

[45] Satoh-Takayama N., Kato T., Motomura Y., Kageyama T., Taguchi-Atarashi N., Kinoshita-Daitoku R., Kuroda E., Di Santo J.P., Mimuro H., Moro K. (2020). Bacteria-Induced Group 2 Innate Lymphoid Cells in the Stomach Provide Immune Protection through Induction of IgA. Immunity.

[46] Aguilera M., Cerdà-Cuéllar M., Martínez V. (2015). Antibiotic-induced dysbiosis alters host-bacterial interactions and leads to colonic sensory and motor changes in mice. Gut Microbes.

[47] Obata T., Goto Y., Kunisawa J., Sato S., Sakamoto M., Setoyama H., Matsuki T., Nonaka K., Shibata N., Gohda M. (2010). Indigenous opportunistic bacteria inhabit mammalian gut-associated lymphoid tissues and share a mucosal antibody-mediated symbiosis. Proc. Natl. Acad. Sci. USA.

[48] Yang C., Mogno I., Contijoch E.J., Borgerding J.N., Aggarwala V., Li Z., Siu S., Grasset E.K., Helmus D.S., Dubinsky M.C. (2020). Fecal IgA Levels Are Determined by Strain-Level Differences in Bacteroides ovatus and Are Modifiable by Gut Microbiota Manipulation. Cell Host Microbe.

[49] Yiu J.H.C., Dorweiler B., Woo C.W. (2016). Interaction between gut microbiota and toll-like receptor: From immunity to metabolism. J. Mol. Med..

[50] Chen Y.-Y., Chen D.-Q., Chen L., Liu J.-R., Vaziri N.D., Guo Y., Zhao Y.-Y. (2019). Microbiome–metabolome reveals the contribution of gut–kidney axis on kidney disease. J. Transl. Med..

[51] Huang Y., Zhou J., Wang S., Xiong J., Chen Y., Liu Y., Xiao T., Li Y., He T., Li Y. (2020). Indoxyl sulfate induces intestinal barrier injury through IRF1-DRP1 axis-mediated mitophagy impairment. Theranostics.

[52] Lau W.L., Savoj J., Nakata M.B., Vaziri N.D. (2018). Altered microbiome in chronic kidney disease: Systemic effects of gut-derived uremic toxins. Clin. Sci..

[53] Song X., Sun X., Oh S.F., Wu M., Zhang Y., Zheng W., Geva-Zatorsky N., Jupp R., Mathis D., Benoist C. (2020). Microbial bile acid metabolites modulate gut RORγ+ regulatory T cell homeostasis. Nature.

[54] Huang J., Pearson J.A., Peng J., Hu Y., Sha S., Xing Y., Huang G., Li X., Hu F., Xie Z. (2020). Gut microbial metabolites alter IgA immunity in type 1 diabetes. J. Clin. Investig..

[55] Luck H., Khan S., Kim J.H., Copeland J.K., Revelo X.S., Tsai S., Chakraborty M., Cheng K., Chan Y.T., Nøhr M.K. (2019). Gut-associated IgA+ immune cells regulate obesity-related insulin resistance. Nat. Commun..

[56] Murphy E.A., Velazquez K.T., Herbert K.M. (2015). Influence of high-fat diet on gut microbiota: A driving force for chronic disease risk. Curr. Opin. Clin. Nutr. Metab. Care.

[57] Papista C., Lechner S., Ben Mkaddem S., LeStang M.-B., Abbad L., Bex-Coudrat J., Pillebout E., Chemouny J.M., Jablonski M., Flamant M. (2015). Gluten exacerbates IgA nephropathy in humanized mice through gliadin–CD89 interaction. Kidney Int..

[58] Coppo R., Roccatello D., Amore A., Quattrocchio G., Molino A., Gianoglio B., Amoroso A., Bajardi P., Piccoli G. (1990). Effects of a gluten-free diet in primary IgA nephropathy. Clin. Nephrol..

[59] Yap H.K., Sakai R.S., Woo K.T., Lim C.H., Jordan S.C. (1987). Detection of bovine serum albumin in the circulating IgA immune complexes of patients with IgA nephropathy. Clin. Immunol. Immunopathol..

[60] Barbour T.D., Pickering M.C., Cook H.T. (2013). Recent insights into C3 glomerulopathy. Nephrol. Dial. Transplant..

[61] Medjeral-Thomas N.R., Lomax-Browne H.J., Beckwith H., Willicombe M., McLean A.G., Brookes P., Pusey C.D., Falchi M., Cook H.T., Pickering M.C. (2017). Circulating complement factor H–related proteins 1 and 5 correlate with disease activity in IgA nephropathy. Kidney Int..

[62] Tortajada A., Gutiérrez E., de Jorge E.G., Anter J., Segarra A., Espinosa M., Blasco M., Roman E., Marco H., Quintana L.F. (2017). Elevated factor H–related protein 1 and factor H pathogenic variants decrease complement regulation in IgA nephropathy. Kidney Int..

[63] Espinosa M., Ortega R., Sánchez M., Segarra A., Salcedo M.T., González F., Camacho R., Valdivia M.A., Cabrera R., López K. (2014). Association of C4d Deposition with Clinical Outcomes in IgA Nephropathy. Clin. J. Am. Soc. Nephrol..

[64] Roos A., Rastaldi M.P., Calvaresi N., Oortwijn B.D., Schlagwein N., Van Gijlswijk-Janssen D.J., Stahl G., Matsushita M., Fujita T., van Kooten C. (2006). Glomerular Activation of the Lectin Pathway of Complement in IgA Nephropathy Is Associated with More Severe Renal Disease. J. Am. Soc. Nephrol..

[65] Medjeral-Thomas N.R., Troldborg A., Constantinou N., Lomax-Browne H.J., Hansen A.G., Willicombe M., Pusey C.D., Cook H.T., Thiel S., Pickering M.C. (2017). Progressive IgA Nephropathy Is Associated With Low Circulating Mannan-Binding Lectin–Associated Serine Protease-3 (MASP-3) and Increased Glomerular Factor H–Related Protein-5 (FHR5) Deposition. Kidney Int. Rep..

[66] Stangou M., Alexopoulos E., Pantzaki A., Leonstini M., Memmos D. (2008). C5b-9 glomerular deposition and tubular alpha3beta1-integrin expression are implicated in the development of chronic lesions and predict renal function outcome in immunoglobulin A nephropathy. Scand. J. Urol. Nephrol..

[67] Ouyang Y., Zhu L., Shi M., Yu S., Jin Y., Wang Z., Ma J., Yang M., Zhang X., Pan X. (2019). A Rare Genetic Defect of MBL2 Increased the Risk for Progression of IgA Nephropathy. Front. Immunol..

[68] Barbour S.J., Reich H.N. (2012). Risk Stratification of Patients With IgA Nephropathy. Am. J. Kidney Dis..

[69] Cattran D.C., Feehally J., Cook H.T., Liu Z.H., Fervenza F.C., Mezzano S.A., Floege J., Nachman P.H., Gipson D.S., Praga M. (2021). Kidney Disease: Improving Global Outcomes (KDIGO) Glomerular Diseases Work Group. KDIGO 2021 Clinical Practice Guideline for the Management of Glomerular Diseases. Kidney Int..

[70] Roberts I.S., Cook H.T., Troyanov S., Alpers C.E., Amore A., Barratt J., Berthoux F., Bonsib S., Bruijn J.A., A Working Group of the International IgA Nephropathy Network and the Renal Pathology Society (2009). The Oxford classification of IgA nephropathy: Pathology definitions, correlations, and reproducibility. Kidney Int..

[71] Cattran D.C., Coppo R., Cook H.T., Feehally J., Roberts I.S., Troyanov S., Alpers C.E., Amore A., Barratt J., A Working Group of the International IgA Nephropathy Network and the Renal Pathology Society (2009). The Oxford classification of IgA nephropathy: Rationale, clinicopathological correlations, and classification. Kidney Int..

[72] Haas M., Verhave J.C., Liu Z.-H., Alpers C.E., Barratt J., Becker J.U., Cattran D., Cook H.T., Coppo R., Feehally J. (2016). A Multicenter Study of the Predictive Value of Crescents in IgA Nephropathy. J. Am. Soc. Nephrol..

[73] Barbour S.J., Coppo R., Zhang H., Liu Z.-H., Suzuki Y., Matsuzaki K., Katafuchi R., Er L., Espino-Hernandez G., Kim S.J. (2019). Evaluating a New International Risk-Prediction Tool in IgA Nephropathy. JAMA Intern. Med..

[74] Barbour S.J., Coppo R., Zhang H., Liu Z.-H., Suzuki Y., Matsuzaki K., Er L., Reich H.N., Barratt J., Cattran D.C. (2022). Application of the International IgA Nephropathy Prediction Tool one or two years post-biopsy. Kidney Int..

[75] Shimozato S., Hiki Y., Odani H., Takahashi K., Yamamoto K., Sugiyama S. (2008). Serum under-galactosylated IgA1 is increased in Japanese patients with IgA nephropathy. Nephrol. Dial. Transplant..

[76] Camilla R., Suzuki H., Daprà V., Loiacono E., Peruzzi L., Amore A., Ghiggeri G.M., Mazzucco G., Scolari F., Gharavi A.G. (2011). Oxidative Stress and Galactose-Deficient IgA1 as Markers of Progression in IgA Nephropathy. Clin. J. Am. Soc. Nephrol..

[77] Zhang K., Li Q., Zhang Y., Shang W., Wei L., Li H., Gao S., Yan T., Jia J., Liu Y. (2019). Clinical Significance of Galactose-Deficient IgA1 by KM55 in Patients with IgA Nephropathy. Kidney Blood Press. Res..

[78] Yanagawa H., Suzuki H., Suzuki Y., Kiryluk K., Gharavi A.G., Matsuoka K., Makita Y., Julian B.A., Novak J., Tomino Y. (2014). A Panel of Serum Biomarkers Differentiates IgA Nephropathy from Other Renal Diseases. PLoS ONE.

[79] Zhao N., Hou P., Lv J., Moldoveanu Z., Li Y., Kiryluk K., Gharavi A.G., Novak J., Zhang H. (2012). The level of galactose-deficient IgA1 in the sera of patients with IgA nephropathy is associated with disease progression. Kidney Int..

[80] Berthoux F., Suzuki H., Thibaudin L., Yanagawa H., Maillard N., Mariat C., Tomino Y., Julian B.A., Novak J. (2012). Autoantibodies Targeting Galactose-Deficient IgA1 Associate with Progression of IgA Nephropathy. J. Am. Soc. Nephrol..

[81] Zhang X., Lv J., Liu P., Xie X., Wang M., Liu D., Zhang H., Jin J. (2021). Poly-IgA Complexes and Disease Severity in IgA Nephropathy. Clin. J. Am. Soc. Nephrol..

[82] Baek H.S., Han M.H., Kim Y.J., Cho M.H. (2018). Clinical Relevance of C4d Deposition in Pediatric Immunoglobulin A Nephropathy. Fetal Pediatr. Pathol..

[83] Marek-Bukowiec K., Konieczny A., Ratajczyk K., Witkiewicz W. (2018). Candidate Urine Peptide Biomarkers for IgA Nephropathy: Where Are We Now?. Dis. Mark..

[84] Rocchetti M.T., Papale M., D’Apollo A.M., Suriano I.V., Di Palma A.M., Vocino G., Montemurno E., Varraso L., Grandaliano G., Di Paolo S. (2013). Association of Urinary Laminin G-Like 3 and Free K Light Chains with Disease Activity and Histological Injury in IgA Nephropathy. Clin. J. Am. Soc. Nephrol..

[85] Zewinger S., Rauen T., Rudnicki M., Federico G., Wagner M., Triem S., Schunk S.J., Petrakis I., Schmit D., Wagenpfeil S. (2018). Dickkopf-3 (DKK3) in Urine Identifies Patients with Short-Term Risk of eGFR Loss. J. Am. Soc. Nephrol..

[86] Wang G., Kwan B.C.-H., Lai F.M.-M., Chow K.-M., Kam-Tao Li P., Szeto C.-C. (2010). Expression of microRNAs in the urinary sediment of patients with IgA nephropathy. Dis. Mark..

[87] Serino G., Sallustio F., Cox S.N., Pesce F., Schena F.P. (2012). Abnormal miR-148b Expression Promotes Aberrant Glycosylation of IgA1 in IgA Nephropathy. J. Am. Soc. Nephrol..

[88] Hu S., Bao H., Xu X., Zhou X., Qin W., Zeng C., Liu Z. (2015). Increased miR-374b promotes cell proliferation and the production of aberrant glycosylated IgA1 in B cells of IgA nephropathy. FEBS Lett..

[89] Yu B., Shi S., Lv J., Liu L., Zhou X., Zhu L., Chen P., Yang H., Wang Z., Wang S. (2022). Rapidly progressive IgA nephropathy: Clinicopathological characteristics and outcomes assessed according to the revised definition of the KDIGO 2021 Guideline. Nephrol. Dial. Transplant..

[90] Floege J., Feehally J. (2013). Treatment of IgA nephropathy and Henoch-Schönlein nephritis. Nat. Rev. Nephrol..

[91] Lennartz D.P., Seikrit C., Wied S., Fitzner C., Eitner F., Hilgers R.-D., Rauen T., Floege J. (2020). Single versus dual blockade of the renin-angiotensin system in patients with IgA nephropathy. J. Nephrol..

[92] Wheeler D.C., Toto R.D., Stefánsson B.V., Jongs N., Chertow G.M., Greene T., Hou F.F., McMurray J.J., Pecoits-Filho R., Correa-Rotter R. (2021). A pre-specified analysis of the DAPA-CKD trial demonstrates the effects of dapagliflozin on major adverse kidney events in patients with IgA nephropathy. Kidney Int..

[93] Rauen T., Eitner F., Fitzner C., Sommerer C., Zeier M., Otte B., Panzer U., Peters H., Benck U., Mertens P.R. (2015). Intensive Supportive Care plus Immunosuppression in IgA Nephropathy. N. Engl. J. Med..

[94] Lv J., Zhang H., Wong M.G., Jardine M.J., Hladunewich M., Jha V., Monaghan H., Zhao M., Barbour S., Reich H. (2017). Effect of Oral Methylprednisolone on Clinical Outcomes in Patients With IgA Nephropathy: The TESTING Randomized Clinical Trial. JAMA.

[95] Wong M.G., Lv J., Hladunewich M.A., Jha V., Hooi L.S., Monaghan H., Zhao M., Barbour S., Reich H.N., Cattran D. (2021). The Therapeutic Evaluation of Steroids in IgA Nephropathy Global (TESTING) Study: Trial Design and Baseline Characteristics. Am. J. Nephrol..

[96] Lv J., Zhang H., Wong M.G., Jardine M.J., Hladunewich M., Jha V., Monaghan H., Zhao M., Barbour S., Reich H. (2022). Effect of Oral Methylprednisolone on Decline in Kidney Function or Kidney Failure in Patients With IgA Nephropathy: The TESTING Randomized Clinical Trial. JAMA.

[97] Tang S., Leung J.C., Chan L.Y., Lui Y.H., Tang C.S., Kan C.H., Ho Y.W., Lai K.N. (2005). Mycophenolate mofetil alleviates persistent proteinuria in IgA nephropathy. Kidney Int..

[98] Tang S.C., Tang A.W., Wong S.S., Leung J.C., Ho Y.W., Lai K.N. (2010). Long-term study of mycophenolate mofetil treatment in IgA nephropathy. Kidney Int..

[99] Floege J., Rauen T., Tang S.C.W. (2021). Current treatment of IgA nephropathy. Semin. Immunopathol..

[100] Makita Y., Suzuki H., Kano T., Takahata A., Julian B.A., Novak J., Suzuki Y. (2019). TLR9 activation induces aberrant IgA glycosylation via APRIL- and IL-6–mediated pathways in IgA nephropathy. Kidney Int..

[101] Torigoe M., Sakata K., Ishii A., Iwata S., Nakayamada S., Tanaka Y. (2018). Hydroxychloroquine efficiently suppresses inflammatory responses of human class-switched memory B cells via Toll-like receptor 9 inhibition. Clin. Immunol..

[102] Liu L.-J., Yang Y.-Z., Shi S.-F., Bao Y.-F., Yang C., Zhu S.-N., Sui G.-L., Chen Y.-Q., Lv J.-C., Zhang H. (2019). Effects of Hydroxychloroquine on Proteinuria in IgA Nephropathy: A Randomized Controlled Trial. Am. J. Kidney Dis..

[103] Gao R., Wu W., Wen Y., Li X. (2017). Hydroxychloroquine alleviates persistent proteinuria in IgA nephropathy. Int. Urol. Nephrol..

[104] Tang C., Lv J.-C., Shi S.-F., Chen Y.-Q., Liu L.-J., Zhang H. (2021). Long-term safety and efficacy of hydroxychloroquine in patients with IgA nephropathy: A single-center experience. J. Nephrol..

[105] Stefan G., Mircescu G. (2021). Hydroxychloroquine in IgA nephropathy: A systematic review. Ren. Fail..

[106] Rollino C., Vischini G., Coppo R. (2016). IgA nephropathy and infections. J. Nephrol..

[107] Takahara M., Nagato T., Nozaki Y., Kumai T., Katada A., Hayashi T., Harabuchi Y. (2019). A proliferation-inducing ligand (APRIL) induced hyper-production of IgA from tonsillar mononuclear cells in patients with IgA nephropathy. Cell. Immunol..

[108] Zheng N., Xie K., Ye H., Dong Y., Wang B., Luo N., Fan J., Tan J., Chen W., Yu X. (2020). TLR7 in B cells promotes renal inflammation and Gd-IgA1 synthesis in IgA nephropathy. J. Clin. Investig..

[109] Myette J.R., Kano T., Suzuki H., Sloan S.E., Szretter K.J., Ramakrishnan B., Adari H., Deotale K.D., Engler F., Shriver Z. (2019). A Proliferation Inducing Ligand (APRIL) targeted antibody is a safe and effective treatment of murine IgA nephropathy. Kidney Int..

[110] Mathur M., Barratt J., Suzuki Y., Engler F., Pasetti M.F., Yarbrough J., Sloan S., Oldach D. (2022). Safety, Tolerability, Pharmacokinetics, and Pharmacodynamics of VIS649 (Sibeprenlimab), an APRIL-Neutralizing IgG2 Monoclonal Antibody, in Healthy Volunteers. Kidney Int. Rep..

[111] Barratt J., Tumlin J., Suzuki Y., Kao A., Aydemir A., Pudota K., Jin H., Gühring H., Appel G. (2022). Randomized Phase II JANUS Study of Atacicept in Patients With IgA Nephropathy and Persistent Proteinuria. Kidney Int. Rep..

[112] Maixnerova D., El Mehdi D., Rizk D.V., Zhang H., Tesar V. (2022). New Treatment Strategies for IgA Nephropathy: Targeting Plasma Cells as the Main Source of Pathogenic Antibodies. J. Clin. Med..

[113] Lafayette R.A., Canetta P., Rovin B.H., Appel G.B., Novak J., Nath K.A., Sethi S., Tumlin J.A., Mehta K., Hogan M. (2016). A Randomized, Controlled Trial of Rituximab in IgA Nephropathy with Proteinuria and Renal Dysfunction. J. Am. Soc. Nephrol..

[114] Lechner S.M., Papista C., Chemouny J.M., Berthelot L., Monteiro R.C. (2015). Role of IgA receptors in the pathogenesis of IgA nephropathy. J. Nephrol..

[115] Jhee J.H., Nam B.Y., Park J.T., Kim H.W., Chang T.I., Kang E.W., Lim B.J., Yoo T.-H., Kang S.-W., Jeong H.J. (2020). CD71 mesangial IgA1 receptor and the progression of IgA nephropathy. Transl. Res..

[116] Moresco R.N., Speeckaert M.M., Zmonarski S.C., Krajewska M., Komuda-Leszek E., Perkowska-Ptasinska A., Gesualdo L., Rocchetti M.T., Delanghe S.E., Vanholder R. (2016). Urinary myeloid IgA Fc alpha receptor (CD89) and transglutaminase-2 as new biomarkers for active IgA nephropathy and henoch-Schönlein purpura nephritis. BBA Clin..

[117] Lamm M.E., Emancipator S.N., Robinson J.K., Yamashita M., Fujioka H., Qiu J., Plaut A.G. (2008). Microbial IgA Protease Removes IgA Immune Complexes from Mouse Glomeruli In Vivo: Potential Therapy for IgA Nephropathy. Am. J. Pathol..

[118] Wang L., Li X., Shen H., Mao N., Wang H., Cui L., Cheng Y., Fan J. (2016). Bacterial IgA protease-mediated degradation of agIgA1 and agIgA1 immune complexes as a potential therapy for IgA Nephropathy. Sci. Rep..

[119] Lechner S.M., Abbad L., Boedec E., Papista C., Le Stang M.-B., Moal C., Maillard J., Jamin A., Bex-Coudrat J., Wang Y. (2016). IgA1 Protease Treatment Reverses Mesangial Deposits and Hematuria in a Model of IgA Nephropathy. J. Am. Soc. Nephrol..

[120] Xie X., Li J., Liu P., Wang M., Gao L., Wan F., Lv J., Zhang H., Jin J. (2022). Chimeric Fusion between *Clostridium Ramosum* IgA Protease and IgG Fc Provides Long-Lasting Clearance of IgA Deposits in Mouse Models of IgA Nephropathy. J. Am. Soc. Nephrol..

[121] Tam F., Tumlin J., Barratt J., Rovin B., Roberts I., Roufosse C., Cook H., Tong S., Magilavy D. (2019). Lafayette. Sun-036 Spleen Tyrosine Kinase (Syk) Inhibition in Iga Nephropathy: A Global, Phase Ii, Randomised Placebo-Controlled Trial of Fostamatinib. Kidney Int. Rep..

[122] McAdoo S., Tam F.W. (2018). Role of the Spleen Tyrosine Kinase Pathway in Driving Inflammation in IgA Nephropathy. Semin. Nephrol..

[123] Yiu W.H., Chan K.W., Chan L.Y.Y., Leung J.C.K., Lai K.N., Tang S.C.W. (2021). Spleen Tyrosine Kinase Inhibition Ameliorates Tubular Inflammation in IgA Nephropathy. Front. Physiol..

[124] Nakata J., Suzuki Y., Suzuki H., Sato D., Kano T., Yanagawa H., Matsuzaki K., Horikoshi S., Novak J., Tomino Y. (2014). Changes in Nephritogenic Serum Galactose-Deficient IgA1 in IgA Nephropathy following Tonsillectomy and Steroid Therapy. PLoS ONE.

[125] Moriyama T., Karasawa K., Miyabe Y., Akiyama K., Iwabuchi Y., Ogura S., Takabe T., Sugiura N., Seki M., Hanafusa N. (2020). Long-Term Beneficial Effects of Tonsillectomy on Patients with Immunoglobulin A Nephropathy. Kidney360.

[126] Hirano K., Matsuzaki K., Yasuda T., Nishikawa M., Yasuda Y., Koike K., Maruyama S., Yokoo T., Matsuo S., Kawamura T. (2019). Association Between Tonsillectomy and Outcomes in Patients With Immunoglobulin A Nephropathy. JAMA Netw. Open.

[127] Bager P., Gørtz S., Feenstra B., Andersen N.N., Jess T., Frisch M., Melbye M. (2019). Increased Risk of Inflammatory Bowel Disease in Families with Tonsillectomy: A Danish National Cohort Study. Epidemiology.

[128] Wu M.-C., Ma K.S.-K., Wang Y.-H., Wei J.C.-C. (2020). Impact of tonsillectomy on irritable bowel syndrome: A nationwide population-based cohort study. PLoS ONE.

[129] Lee M., Suzuki H., Kato R., Nakayama M., Fukao Y., Kano T., Makita Y., Kobayashi T., Sato D., Kihara M. Study Protocol for Validation of the Safety and Efficacy of Topical Steroid Therapy Targeting Nasal-Associated Lymphoid Tissue in IgA Nephropathy: A Single-Centre, Open-Label, Historical Controlled Study. Research Square.

[130] Coppo R. (2018). The Gut-Renal Connection in IgA Nephropathy. Semin. Nephrol..

[131] Barratt J., Lafayette R., Kristensen J., Stone A., Cattran D., Floege J., Tesar V., Trimarchi H., Zhang H., Eren N. (2022). Results from part A of the multi-center, double-blind, randomized, placebo-controlled NefIgArd trial, which evaluated targeted-release formulation of budesonide for the treatment of primary immunoglobulin A nephropathy. Kidney Int..

[132] Fellström B.C., Barratt J., Cook H., Coppo R., Feehally J., de Fijter J.W., Floege J., Hetzel G., Jardine A.G., Locatelli F. (2017). Targeted-release budesonide versus placebo in patients with IgA nephropathy (NEFIGAN): A double-blind, randomised, placebo-controlled phase 2b trial. Lancet.

[133] Zhang W., Yuan Y., Li X., Luo J., Zhou Z., Yu L., Wang G. (2022). Orange-derived and dexamethasone-encapsulated extracellular vesicles reduced proteinuria and alleviated pathological lesions in IgA nephropathy by targeting intestinal lymphocytes. Front. Immunol..

[134] Zhong Z., Tan J., Tan L., Tang Y., Qiu Z., Pei G., Qin W. (2020). Modifications of gut microbiota are associated with the severity of IgA nephropathy in the Chinese population. Int. Immunopharmacol..

[135] He J.-W., Zhou X.-J., Li Y.-F., Wang Y.-N., Liu L.-J., Shi S.-F., Xin X.-H., Li R.-S., Falchi M., Lv J.-C. (2021). Associations of Genetic Variants Contributing to Gut Microbiota Composition in Immunoglobin A Nephropathy. Msystems.

[136] Zhao J., Bai M., Ning X., Qin Y., Wang Y., Yu Z., Dong R., Zhang Y., Sun S. (2022). Expansion of *Escherichia-Shigella* in Gut Is Associated with the Onset and Response to Immunosuppressive Therapy of IgA Nephropathy. J. Am. Soc. Nephrol..

[137] Chemouny J.M., Gleeson P.J., Abbad L., Lauriero G., Boedec E., Le Roux K., Monot C., Bredel M., Bex-Coudrat J., Sannier A. (2018). Modulation of the microbiota by oral antibiotics treats immunoglobulin A nephropathy in humanized mice. Nephrol. Dial. Transplant..

[138] Di Leo V., Gleeson P.J., Sallustio F., Bounaix C., Da Silva J., Loreto G., Ben Mkaddem S., Monteiro R.C. (2021). Rifaximin as a Potential Treatment for IgA Nephropathy in a Humanized Mice Model. J. Pers. Med..

[139] Tan J., Dong L., Jiang Z., Tan L., Luo X., Pei G., Qin A., Zhong Z., Liu X., Tang Y. (2022). Probiotics ameliorate IgA nephropathy by improving gut dysbiosis and blunting NLRP3 signaling. J. Transl. Med..

[140] Chai L., Luo Q., Cai K., Wang K., Xu B. (2021). Reduced fecal short-chain fatty acids levels and the relationship with gut microbiota in IgA nephropathy. BMC Nephrol..

[141] Lauriero G., Abbad L., Vacca M., Celano G., Chemouny J.M., Calasso M., Berthelot L., Gesualdo L., De Angelis M., Monteiro R.C. (2021). Fecal Microbiota Transplantation Modulates Renal Phenotype in the Humanized Mouse Model of IgA Nephropathy. Front. Immunol..

[142] Bruchfeld A., Magin H., Nachman P., Parikh S., Lafayette R., Potarca A., Miao S., Bekker P. (2022). C5a receptor inhibitor avacopan in immunoglobulin A nephropathy—An open-label pilot study. Clin. Kidney J..

[143] Barratt J., Rovin B., Zhang H., Kashihara N., Maes B., Rizk D., Trimarchi H., Sprangers B., Meier M., Kollins D. (2022). Pos-546 Efficacy and Safety of Iptacopan in Iga Nephropathy: Results of a Randomized Double-Blind Placebo-Controlled Phase 2 Study at 6 Months. Kidney Int. Rep..

[144] Lafayette R.A., Rovin B.H., Reich H.N., Tumlin J.A., Floege J., Barratt J. (2020). Safety, Tolerability and Efficacy of Narsoplimab, a Novel MASP-2 Inhibitor for the Treatment of IgA Nephropathy. Kidney Int. Rep..

